# Mobile device-based Bluetooth Low Energy Database for range estimation in indoor environments

**DOI:** 10.1038/s41597-022-01406-2

**Published:** 2022-06-08

**Authors:** Pavel Pascacio, Joaquín Torres-Sospedra, Antonio R. Jiménez, Sven Casteleyn

**Affiliations:** 1 Institute of New Imaging Technologies, Universitat Jaume I, Castellón, 12071 Spain; 2 Electrical Engineering Unit, Tampere University, Tampere, 33720 Finland; 3 Centro ALGORITMI, Universidade do Minho, 4800-058 Guimarães, Portugal; 4 Center for Automation and Robotics, Spanish National Research Council (CSIC-UPM), 28500 Madrid, Spain

**Keywords:** Scientific data, Computer science

## Abstract

The demand to enhance distance estimation and location accuracy in a variety of Non-Line-of-Sight (NLOS) indoor environments has boosted investigation into infrastructure-less ranging and collaborative positioning approaches. Unfortunately, capturing the required measurements to support such systems is tedious and time-consuming, as it requires simultaneous measurements using multiple mobile devices, and no such database are available in literature. This article presents a Bluetooth Low Energy (BLE) database, including Received-Signal-Strength (RSS) and Ground-Truth (GT) positions, for indoor positioning and ranging applications, using mobile devices as transmitters and receivers. The database is composed of three subsets: one devoted to the calibration in an indoor scenario; one for ranging and collaborative positioning under Non-Line-of-Sight conditions; and one for ranging and collaborative positioning in real office conditions. As a validation of the dataset, a baseline analysis for data visualization, data filtering and collaborative distance estimation applying a path-loss based on the Levenberg-Marquardt Least Squares Trilateration method are included.

## Abstract (summary)

**Table:** 

Measurement(s)	Received Signal Strength of Bluetooth Low Energy
Technology Type(s)	Bluetooth Low Energy
Factor Type(s)	position
Sample Characteristic - Organism	Mobile devices
Sample Characteristic - Environment	Indoor enviroments

## Background & Summary

In recent years, the technological progress and the growing interest in infrastructure-less positioning systems has boosted the development of systems based on wearables for location-based service (LBS) applications. Despite the diversity of indoor positioning technologies, Bluetooth Low Energy (BLE) is widely used because it is a core communications technology in mobile devices, has a low energy profile, is not expensive, and does not require additional expensive hardware for positioning purposes. In fact, BLE is a straightforward technology to implement RSS-based positioning approaches^1–5^ using, for instance, the iBeacon protocol^5–7^. In addition, over the last year, it has also played a key role as part of COVID-19 ranging-based contact-tracing applications^8^, where the distance between devices needs to be estimated under various conditions.

However, wireless signals are susceptible to attenuation, interference, and multipath propagation due to Non-line-of-sight (NLOS), environment geometries, presence of crowds, and other sources of noise^8–10^. These phenomena induce a random variation on the RSS and therefore a fluctuation on the position estimation^8^. Among ranging and positioning approaches, RSS-based approaches (e.g., proximity, ranging and fingerprinting) are the most affected by this drawback^4^.

**Fig. 1:**
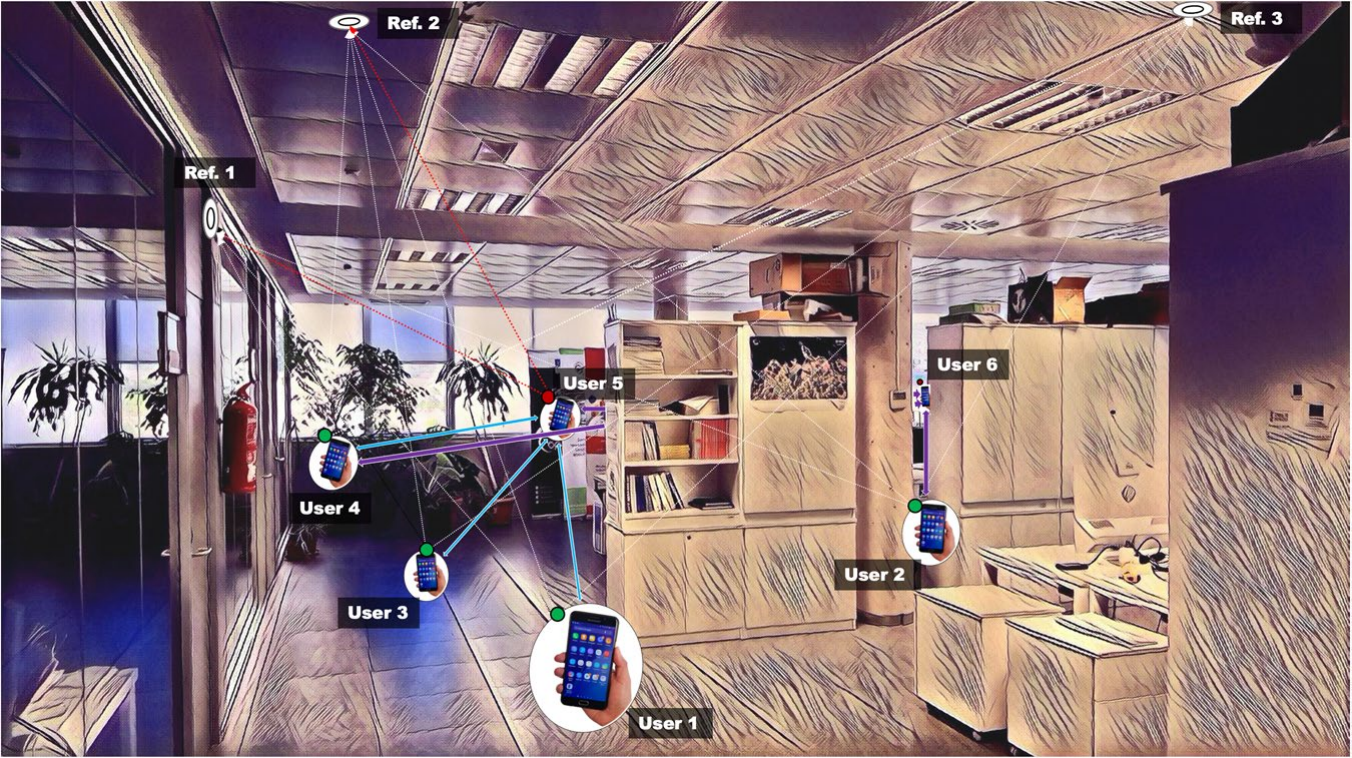
Example of a Collaborative IPS scenario based on BLE and smartphones.

Currently, smartphones are ubiquitous and the density of smartphone users within a small area is increasing, which has been exploited to develop and/or improve user-to-user(s) distance estimations and collaborative Indoor Position Systems (IPSs). In the latter case, neighboring devices are used to estimate or improve their position by exchanging their estimated absolute localization and measuring the relative distance between them^11^. Figure. 1 illustrates a collaborative IPS. In this scenario, User 6 (behind the bookshelves) cannot accurately estimate his position because the bookshelves block the LOS with respect to the three anchors references (Ref. 1-3), so, the surrounding users (1–5) collaborate to improve User 6’s position by broadcasting their position using BLE messages. To realise such collaborative systems, it is crucial to study, characterize and calibrate the RSS under different circumstances and through diverse devices with the purpose of guaranteeing an accurate position estimation and ensuring the robustness of the collaborative IPS. On the other hand, research towards distance estimation (i.e proximity) applications – mainly based on BLE – received an enormous impulse with the COVID-19 crisis, due to its use in (indoor) contact-tracing apps. However, similar problems as in indoor positioning systems are reported, namely a high amount of false positives due to several factors in the indoor environment, such as NLOS conditions, interference between several devices’ signals and varying transmission patterns^12^.

Despite the fact that the scientific community has exhaustively evaluated new positioning and ranging algorithms through mathematical models, evaluations with real empirical data–as a result of experiments carried out in various scenarios, devices, and conditions–are necessary to understand their real-world behavior. Nevertheless, data collection in such scenarios is a time-intensive activity, requiring cumbersome setups with multiple mobile devices. Therefore, sharing the databases with a detailed methodology of the data collection procedures, tests carried out and other relevant aspects for their interpretation is a valuable contribution for researchers who are unable to perform their own experiments. Furthermore, it is a good research practice, as it allows researchers to evaluate their solutions in a different context or scenario, promoting the generalization of obtained results. Finally, the re-use of such datasets enables research reproducibility of the proposed methods, as the community can reproduce the experiments and verify the results using the same data.

The number of published databases related to BLE and RSS solely based on smartphones, in both transmission and reception, is almost nil^1^. Nevertheless, they do not consider the transmission and reception from multiple smartphones. Most of the databases are focused on fingerprinting systems where commercial BLE beacons or other ad-hoc devices mounted in building structures are used as transmitters. On the other side, the receivers are usually smartphones or other wearable devices^4,13,14^. Within the few available databases based only on smartphones, one of them was developed within the framework of a contact-tracing smartphone application using BLE^1^. To the best of our knowledge, no publicly available RSS database based on BLE, based solely on data sent and received with a diverse set of multiple smartphones, exists.

The aim of this paper is to share with the scientific community an RSS database based on BLE, which considers and provides all the necessary data for device-to-device(s) ranging and collaborative indoor positioning systems based on mobile devices. The database consist of three different subsets, one for calibration purposes, and two with real-life distributions. The subsets are outline as follows:

- **Subset-A “Calibration in Line-of-sight (LOS)”:** Data for calibrating the relation in LOS between the RSS and distance for six mobile devices (five smartphones and one tablet) considering one indoor environment. Measurements are collected considering 12 reference points located every meter in a straight line on the floor.
- **Subset-B “Ranging and Collaborative positioning with blocking of LOS, due to deliberate and frequent walking in the environment”:** Data for ranging and collaborative positioning from five static mobile devices (also used in Subset-A) in four set-ups with frequent and intentional walks around the indoor environment of one person, causing various NLOS conditions.
- **Subset-C “Ranging and Collaborative positioning in real office conditions”:** Data for ranging and collaborative positioning from five static mobile devices in seven set-ups in normal office conditions (i.e. people sitting at their desk and sporadically walking around the environments doing regular office tasks).

Moreover, we systematically describe the data collection process, post-processing and sharing formats to allow other researchers involved in indoor positioning to extend the proposed database with other relevant scenarios and environments.

Finally, we present the technical validation of the proposed database. The first validation illustrates how to process the raw BLE signals to estimate the distance between two devices using the Logarithmic Path-loss model. The second validation is devoted to assess the use of BLE signals for collaborative positioning, which includes device-to-device ranging.

## Methods

### Hardware and Software for BLE advertising and data collection procedure

The hardware used for the BLE advertising and data collection in the experiments consists of six mobile devices. The mobiles’ names, sorted by ID number, are Galaxy S8 (01), Lenovo Yoga Book (02), Galaxy A7 Duos (03), Galaxy S6 (04), Honor 20 Lite (05), and Galaxy A5 (06). Relevant information of each smartphone is detailed in Table 1.

**Table 1:** Description of mobile devices.

ID	Mobile name	Model	Brand	Bluetooth version	Android version	API version
01	Galaxy S8	SM-G950F	Samsung	5	9	28
02	Lenovo Yoga Book	Lenovo YB1-X90F	Lenovo	4.0	6.0.1	23
03	Galaxy A7 Duos	SM-A7100	Samsung	4.1	7.1.1	25
04	Galaxy S6	SM-G920F	Samsung	4.1	7	24
05	Honor 20 Lite	HRY-LX1T	Huawei	4.2	9	28
06	Galaxy A5	SM-A500FU	Samsung	4.0	6.0.1	23

The software used for the experiments is based on the *GetSensorData* Android application^15^, which allows to collect and save information from smartphone sensors and wireless communications into *logfiles* (text files with comma separated format and TXT extension). However, since it was originally designed for data collection, it does not have the BLE advertise mode feature. Therefore, we extended the *GetSensorData* application with the BLE advertising feature, enabling us to broadcast advertisements using the iBeacon protocol designed by Apple, which is one of the most used by mobile devices. The iBeacon protocol enables mobile devices, located within a short-range, to broadcast and receive information through BLE packets^7^. The iBeacon (BLE) advertisement period was set to 100 ms (i.e., 10 Hz). Subsequently, the modified application was installed in each of the smartphones. It should be noted that BLE is supported since Android version 4.3, nevertheless, the transmission of BLE beacons is only available since Android version 5.0 (LOLLIPOP) with API 21^16^. The application is available at the GitLab repository^17^.

**Fig. 2:**
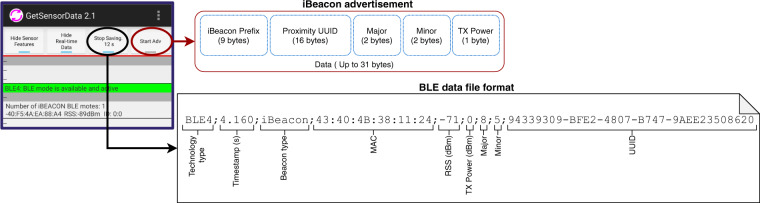
Software used for advertising, scanning and storing BLE beacons.

Figure 2 shows the user interface of the modified *GetSensorData* application installed on the smartphones. The save sensor data and advertise BLE beacon buttons are on the top right corner of the interface, circled in black and red respectively. The save sensor data button serves to save the information of the received BLE packets, together with the information of others internal sensors and wireless communications of the device (e.g, accelerometer, magnetometer, gyroscope, Wi-Fi and GNSS among many others). Specifically, the BLE data file format is composed of the type of technology, in our case BLE; timestamp of the recorded data; type of beacon registered; Media Access Control (MAC) address; RSSs measured; transmission power; Major; Minor and UUID. When the BLE beacon button is enabled, the app starts to broadcast the BLE packets to all surrounding devices. The iBeacon advertisement packets’ structure is composed of five elements as shown in Fig. 2: iBeacon Prefix; Proximity UUID; Major; Minor and TX Power (indicating the signal strength one meter from the device), of which UUID, Major, and Minor are used to identify the device that broadcast the advertisement. The aim of the three type of device identifiers is to provide diverse levels of abstraction to identify and classify the mobile devices within a network infrastructure (e.g., UUID to classify devices belonging to a building, Major to classify them by floor, and Minor to identify each of them within the floor). Additionally, a sixth element, the RSS, is filled by the receiver with the received signal strength in dBm. The TX power value of each device was obtained experimentally, considering the average of the RSS values measured at 1 meter and in LOS from the transmitting mobile. The configuration data for the mobile devices involved in the data collection procedures are detailed in Table 2.

**Table 2:** iBeacon identifiers of each mobile device.

ID	Mobile name	UUID	Major	Minor
01	Galaxy S8	94339309-BFE2-4807-B747-9AEE23508620	8	1
02	Lenovo Yoga Book	94339309-BFE2-4807-B747-9AEE23508620	8	2
03	Galaxy A7 Duos	94339309-BFE2-4807-B747-9AEE23508620	8	3
04	Galaxy S6	94339309-BFE2-4807-B747-9AEE23508620	8	4
05	Honor 20 Lite	94339309-BFE2-4807-B747-9AEE23508620	8	5
06	Galaxy A5	94339309-BFE2-4807-B747-9AEE23508620	8	6

**Fig. 3:**
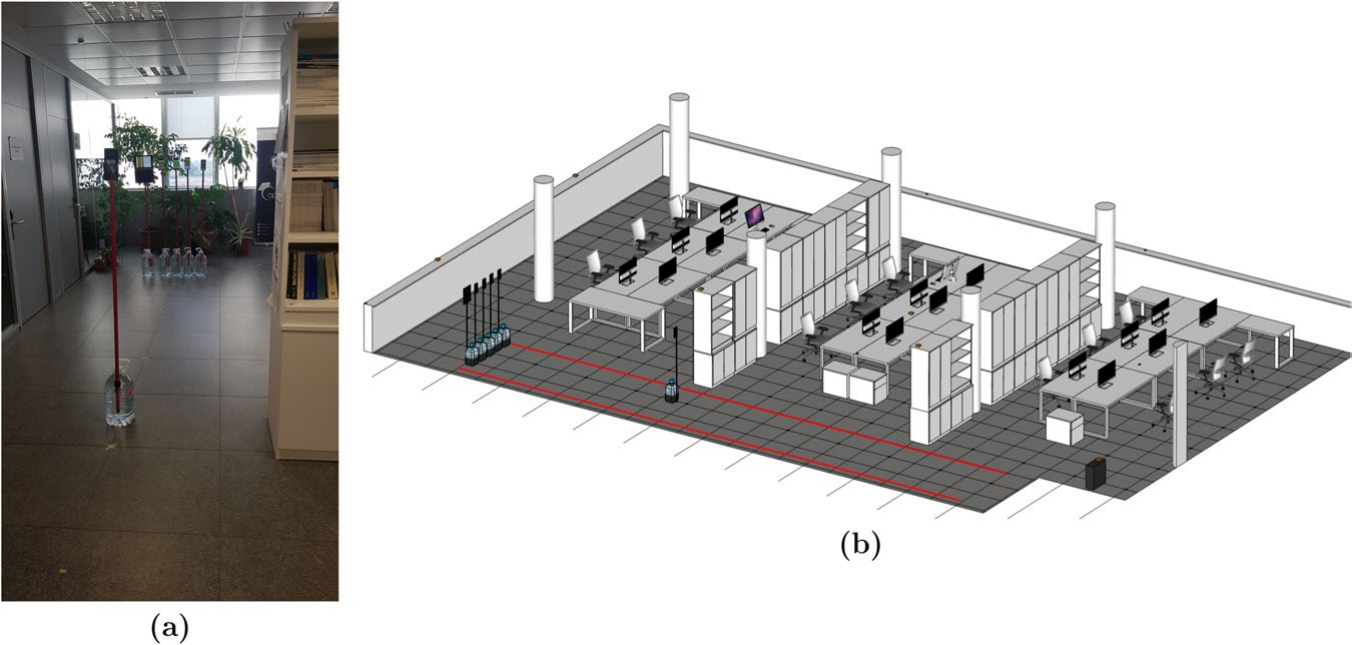
Example of the setup for the Subset-A. (**a**) Real representation. (**b**) 3D model of distribution of the emitters and received in the office.

Each mobile device used during the tests was mounted on a separate pole, 1.5 m from the ground in portrait orientation, in order to avoid interference with radio frequency signals (see Fig. 3(a)).

### Selected scenario for the data collections

We have conducted all the empirical experiments to collect the three subsets (Subset-A, Subset-B and Subset-C) in the research team’s office located at University Jaume I (Castellón, Spain).

The office has an approximate surface area of 10.76 m by 16.71 m and was already illustrated in Fig. 1. The office mainly consists of 14 bookcases, 7 concrete columns, and 3 office work sections equipped with desks, chairs, and computers. This location has already been used in previous data sets for indoor positioning with Wi-Fi fingerprinting, magnetic fields, regular BLE and, even, sensor fusion^13,18,19^.

### Configuration for the subset-A data collection

The data collection of Subset-A was devoted to identifying the BLE signal behavior and calibrating the mobile devices in full Line-of-sight (LOS) conditions in an indoor environment, i.e., the office scenario previously mentioned (see Figs. 1, 3(b)).

We set 12 reference locations every 1 m in a straight line on the floor of the main corridor, from the initial position at 0 m to the last point at 11 m, in LOS. Thus, the degradation of the signal strength can be measured at the reference points to, for instance, tune a propagation model or calibrate a relative distance estimator. This configuration is illustrated in Fig. 3(b), where the red lines delimit the area where the devices under empirical data collection were located.

We placed five of the six used devices (see Table 1) horizontally aligned on the initial position, at 0 m acting as transmitters. The remaining device, was placed consecutively at the remaining reference points at 1 m to 11 m acting as the receiver. The previous procedure was repeated six times in order to allow every device to act as receiver.

Figure 3(a) exemplifies the case where the transmitter devices are horizontally aligned near to the plants (initial position at 0 m) and the receiver is at 4 m.

For each individual data collection, 90 s of raw data from all the sensors (including iBeacon –BLE– advertisements) were recorded with *GetSensorsData* in the receiver mobile device, which was saved into a *logfile*. Thus, we generated a total of 66 *logfile* with raw data, one per emitter and reference location in 1 m to 11 m. In the end, since each of the 66 *logfiles* contains data from 5 different transmitters, 330 ranging emitter-to-receiver pairs of 90 seconds were recorded.

In addition, during the measurements, we ensure that the battery of the devices was not less than 80% of its capacity, we stayed away from the devices and always stood in the same place.

### Scenario and configurations for the subset-B and subset-C data collection

For the collection of Subset-B and Subset-C, we considered different set-ups in the office scenario involving five mobile devices. In contrast to the Subset-A, which was conceived for BLE calibration purposes, Subset-B and Subset-C resemble realistic situations and aim to assess the feasibility of ranging and collaborative positioning using mobile devices and BLE in real-world situations.

For this purpose, different device arrangements with the five devices and various strategies to interfere with LOS among them are implemented. Thus, we provide a greater variety and complexity of test conditions for ranging and collaborative positioning approaches. Within these strategies, we include the modification of the number of occupants in the office, the frequency with which people walk and obstruct the LOS between devices, and the use of diverse fixed obstacles. In each configuration, each smartphone simultaneously broadcasts its own iBeacon (BLE) advertisement, and reads/saves the RSS of the received advertisements broadcasted by the other four devices.

**Fig. 4:**
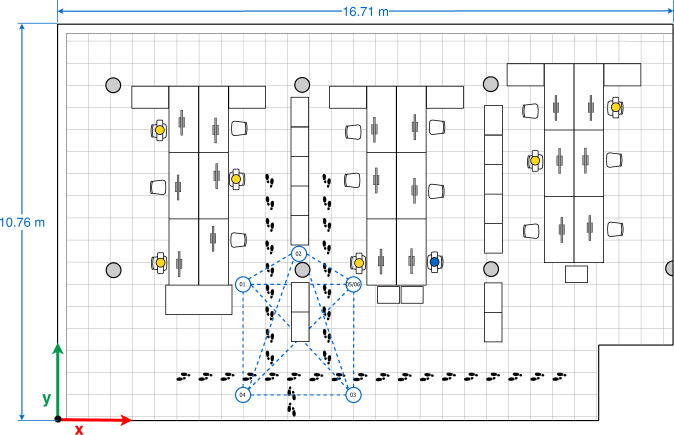
First multi-device configuration.

**Fig. 5:**
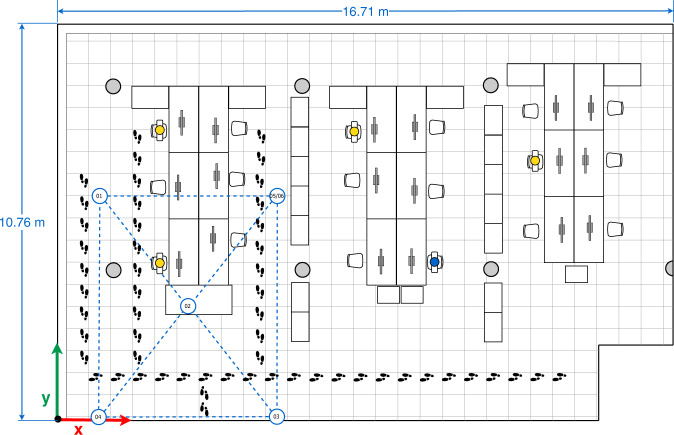
Second multi-device configuration.

**Fig. 6:**
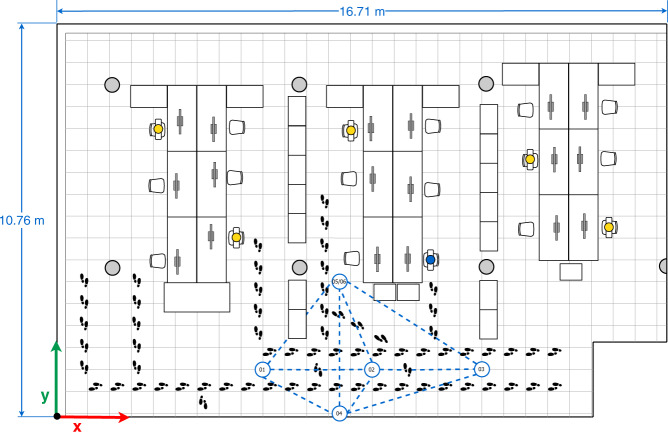
Third multi-device configuration.

**Fig. 7:**
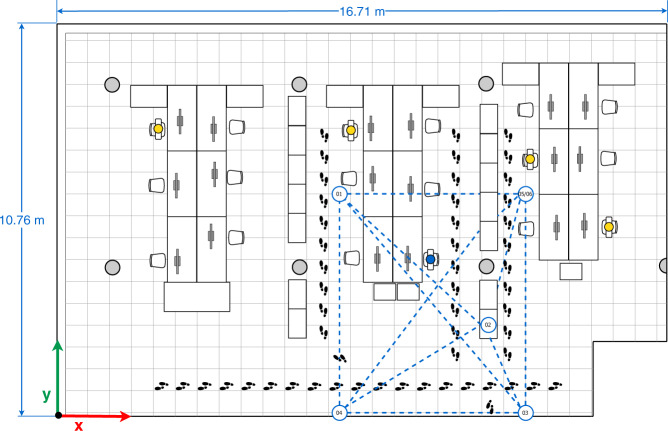
Fourth multi-device configuration.

In the data collection of Subset-B, we consider four arrangements (configurations) made up of five devices, Galaxy S8 (01), Lenovo Yoga Book (02), Galaxy A7 Duos (03) Galaxy S6 (04), Honor Lite (05). These four configurations are illustrated in Figs. 4–7.

In the four configurations, only one person is present in the office who sits in front of the computer (blue person icon on the sketches) or intentionally walks around the office (footprints icons on the sketches) obstructing the LOS path signal between devices. The full description of the configurations is as follows:

- The first multi-device configuration (see Fig. 4) represents five devices exchanging iBeacon advertisements, however, the LOS path signal between the device pairs 01&03, 01&05, and 05&04 are blocked by two wooden bookcases and the pair 02&03 by a concrete column.
- In the second multi-device configuration (see Fig. 5), the device 02 on the desk is located equidistantly from devices 01 and 03, and 04 and 05 respectively. The LOS path signal of the device pairs 03&02, 03&04, 03&05 and 02&04 are not obstructed by any fixed office furniture. Nevertheless, between the device pairs 01&02, 01&05 and 02&05 a set of desks are located.
- The third multi-device configuration (see Fig. 6) represents an arrangement in which one of the devices (device 02) blocks the LOS path signal between devices 01 and 03. Furthermore, the LOS path of the device pairs 01&05 is blocked by a wooden bookcase.
- In the fourth multi-device configuration (see Fig. 7) device 02 is located on one of the shelves of a wooden bookcase, which block its LOS path signal to devices 03 and 05, while devices 01 and 04 maintain LOS conditions with device 02. Also, the device pairs 01&04, 03&04, and 03&05 are in LOS, but the pairs 01&05, 04&05 are not in LOS. Between the device pairs 01&02 and 01&03 a set of desks is located.

**Fig. 8:**
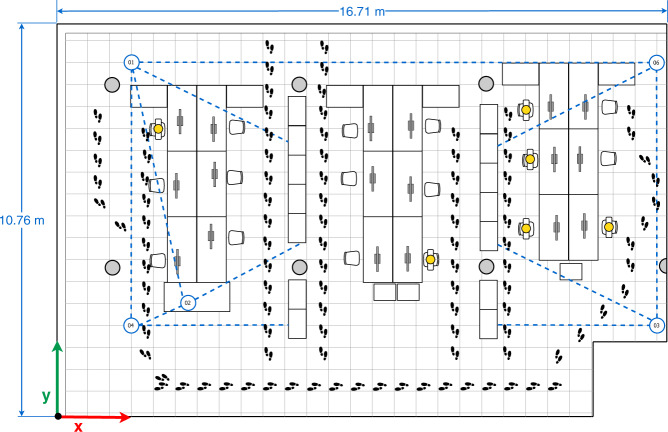
Fifth multi-device configuration.

**Fig. 9:**
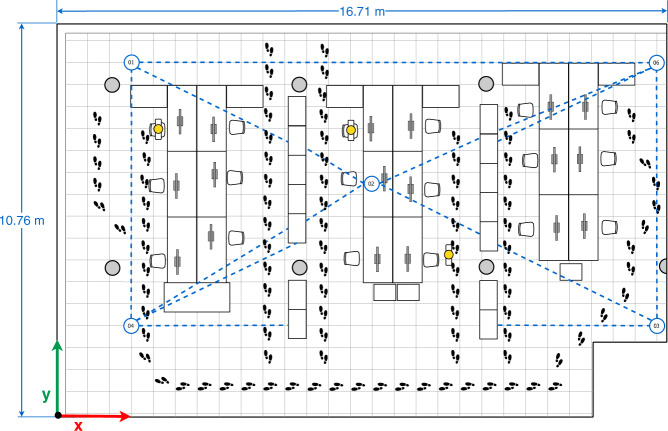
Sixth multi-device configuration.

**Fig. 10:**
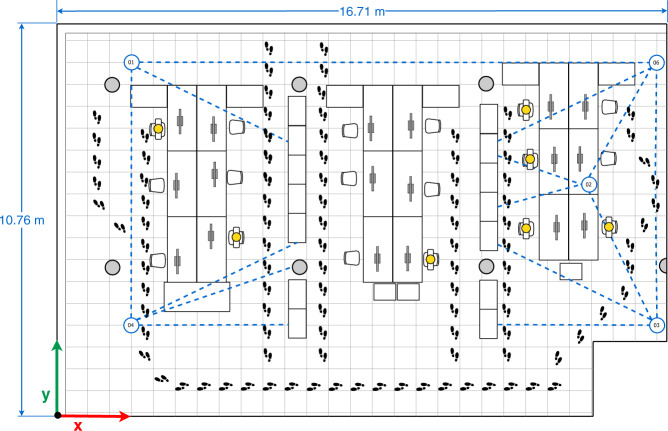
Seventh multi-device configuration.

The data collection of Subset-C was carried out considering seven configurations (see Figs. 4–10) and five devices, i.e. the Galaxy S8 (01), Lenovo Yoga Book (02), Galaxy A7 Duos (03) Galaxy S6 (04), Galaxy A5 (06). We replaced the Honor 20 lite smartphone (05) with a Galaxy A5 (06).

Regarding the seven configurations in the data collection of Subset-C, the first four configurations are similar to the ones reported in Figs. 4–7 for Subset-B, yet the number of people occupying and walking in the office varied (orange person icons plus the blue one on the sketches). In the first configuration 7 people occupied the office; in the second, 5 people; in the third, 6 people; and in the fourth 5 people. The aim of those modifications is to represent the common behavior of workers in the office and observe how they affect the transmission and reception of the signals.

In the fifth, sixth and seventh multi-device configurations the mobile devices 01, 03, 04 and 06 are located in the same location, near to the office corners, as can be seen in the sketches of Figs. 8–10. The main difference among those configurations is the location of mobile device 02. Considering only the four devices in the corners, we can observe that the device pairs 01&06, 01&04, and 03&06 are in LOS, whereas the pairs 01&03, 03&04, and 04&06 present NLOS because of the central bookcases. In particular:

- The fifth multi-device configuration (see Fig. 8) arranges device 02 in LOS with devices 01 and 04, and closer to the device 04. Also, the LOS between device 02 and the devices 03 and 06 is blocked by central bookcases.
- In the sixth multi-device configuration (see Fig. 9), device 02 is located between the central bookcases in order to block its LOS with the devices placed on the corners.
- The seventh multi-device configuration (see Fig. 10) considers the case in which device 02 is in LOS with the devices 03 and 06 and in NLOS with the devices 01 and 04 due to central bookcases.

For Subset-B and Subset-C, raw data was collected with *GetSensorsData* for a period 2 hours in every set-up (configuration). All the devices simultaneously broadcasted their own iBeacon (BLE) advertisement and saved the information from all sensors (including the detected iBeacon advertisements and their RSS). Thus, Subset-B is composed by 20 *logfiles*, whereas Subset-C is composed by 35 *logfiles*. During the duration of the measurements, the line of sight between the devices was altered by the people walking around the office, following the path depicted in each of the multi-device configuration sketches (see Figs. 4–10). Although the desks located between the mobile devices do not fully block the LOS, they partially interfere with the signal propagation. It should be noted that the information storage of each device is not synchronized. However, the time lag - in the order of seconds - should not represent a great inconvenience for static references.

Regarding the valid BLE measurements collected, the average values with standard deviation are reported in Table 4 for the six smartphones in the 90 s windows from Subset-A, the 2 h windows from Subset-B and the 2 h windows from Subset-C. The BLE measurements emitted from tags, beacons and other electrical devices have been discarded, thus only the BLE advertisements emitted from the smartphones reported in Table 3 are considered valid. For the three subsets, an average (with standard deviation) of 1995 ± 731, 79121 ± 54443 and 97371 ± 30650 valid BLE measurements were recorded respectively. It is worth noting that the amount of BLE advertisements received from a particular device depends on the emitter-receiver distance, LOS/NLOS conditions and the BLE chipset at the receiver side. For the later case, Table 4 shows that the Galaxy A5 is only receiving ≈2 BLE messages per receiver and second for the calibration collection in Subset-A, whereas the Galaxy S8 is receiving ≈2 BLE messages per receiver and second (×3 higher) in the same subset.

**Table 3:** Devices’ Ground truth in each configuration of subset A and B.

ID	Ground truth (m)														Subset
	Config. 1		Config. 2		Config. 3		Config. 4		Config. 5		Config. 6		Config. 7		
	x	y	x	y	x	y	x	y	x	y	x	y	x	y	
01	5.05	3.7	1.33	6.1	6.93	1.3	7.75	6.1	2.05	9.7	2.05	9.7	2.05	9.7	B&C
02	6.55	4.55	4.49	3.05	9.93	1.3	11.75	2.75	3.6	3.3	8.7	6.4	14.66	6.45	B&C
03	8.05	0.7	7.66	0.1	12.93	1.3	12.75	0.1	16.45	2.5	16.45	2.5	16.45	2.5	B&C
04	5.05	0.7	1.33	0.1	9.03	0.1	7.75	0.1	2.05	2.5	2.05	2.5	2.05	2.5	B&C
05	8.05	3.7	7.66	6.1	9.03	3.7	12.75	6.1	16.45	9.7	16.45	9.7	16.4	9.7	B
06	8.05	3.7	7.66	6.1	9.03	3.7	12.75	6.1	16.45	9.7	16.45	9.7	16.4	9.7	C

**Table 4:** Average of BLE measurements collected by device in each 90 s and 2 hours time windows.

ID	Mobile name	Subset-A	Subset-B	Subset-C
		(90 s time window)	(2 h time window)	(2 h time window)
01	Galaxy S8	2776 ± 63	80695 ± 60174	81100 ± 32178
02	Lenovo Yoga Book	2604 ± 57	117095 ± 67440	135738 ± 9090
03	Galaxy A7 Duos	1389 ± 188	62281 ± 35708	77029 ± 6454
04	Galaxy S6	2309 ± 127	100933 ± 57994	121047 ± 11955
05	Honor 20 Lite	2004 ± 136	34602 ± 19596	—
06	Galaxy A5	887 ± 99	—	71941 ± 10164

## Data Records

**Fig. 11:**
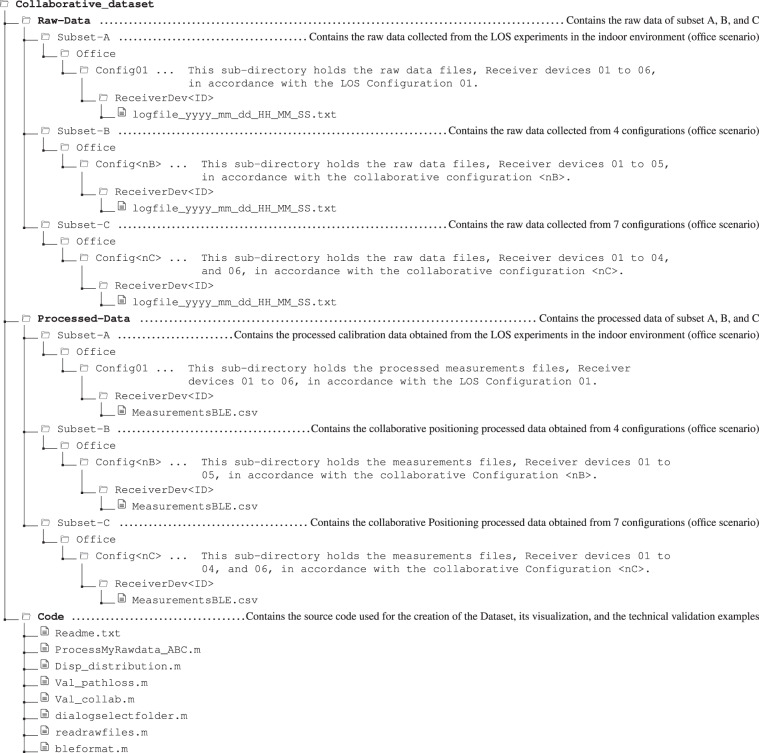
Directory tree of the Database. **<nB>** **∈{01,…,04}** and **<nC>** **∈{01,…,07}**.

Figure 11 presents the directory tree and file structure of the multi-device BLE-RSS database. The database is composed of three main sub-directories, **Raw-Data, Processed-Data**, and **Code**, which is available at the Zenodo repository^20^. The raw data include all the data collected with the *GetSensorsData* application, whereas the processed data just include the required BLE data for ranging and collaborative positioning and they were obtaining after processing the raw data with the scripts included.

The **Raw-Data** contains the raw data corresponding to the three subsets A, B, and C described in Section Methods grouped in sub-directories **Subset-A, Subset-B**, and **Subset-C** respectively. Each of these sub-directories is structured in the same way, containing a sub-directory for each scenario. Currently, the three subsets only considers one of the office scenario, being all the collected data within **Office** folder. Each scenario directory subsequently contains one or more sub-directories which each correspond with a configuration. Finally, each configuration directory contains sub-directories for each receiver device used to collect the data, which contain the raw data files from the independent data collections performed. For instance, Rawdata-A/Office/Config01/ReceiverDev01/*.txt contains the raw data of subset A, office scenario, configuration Config01 and collected with device 01.

The **Processed-Data** directory contains the BLE data and it is structured in the same way. With nested folders to represent the subset, scenario, configuration and device. The processed data from the previous example is included in **Processed-Data/Office/Config01/ReceiverDev01/MeasurementsBLE.csv**. In contrast to the files for the raw data, the processed data files are stored as a single CSV file for each combination of receiver, configuration, scenario and subset.

The **Code** directory contains the Matlab script files for processing the raw data, generate the processed data, visualize useful information, and perform the technical validations. Although in its current form the database focuses on a single scenario (i.e., the office) and several configurations (one for Subset-A, four for Subset-B and Seven for Subset-C), the database has been designed to be easily extended using the proposed hierarchy of folders.

The sub-folders are described in detail as follows. For the raw data (**Raw-Data**):

- Subset-A is composed of 1 scenario (**Office**), 1 configuration (**Config01**) and 6 receiver devices (**ReceiverDev01** to **ReceiverDev06**. A total of 66 TXT raw data files, collected by devices 01 to 06 according to Configuration 01 in the office scenario, are stored in the nested sub-directories **ReceiverDev01** to **ReceiverDev06** (11 files in each sub-directory). For a particular receiver, each of the 11 raw files contain the measurements gathered by the receiver device at a specific reference point. The raw data files, alphabetically sorted, correspond to the data collection with distances between emitter and receiver in the range of 1 m to 11 m. If the data is sequentially collected, starting at 1 m and ending in 11 m, there is no need to alter the *logfiles* file name.
- **Subset-B** is composed of 1 scenario (**Office**) and 4 configurations (**Config01** to **Config04**) and 5 receiver devices (**ReceiverDev01** to **ReceiverDev05**). In each of these nested folders **ReceiverDev01** to **ReceiverDev05**, 1 TXT raw data file is stored containing the raw data of the corresponding receiver device (indicated by the sub-folder name). In total, 20 TXT raw data files are stored.
- **Subset-C** follows the same structure of **Subset-B** sub-directory, but applied to its 7 configurations (**Config01 to Config07**) and 5 receiver devices (**ReceiverDev01** to and **ReceiverDev06**) sub-directories. A total of 35 TXT raw data files are stored.

For the processed data (**Processed-Data**):

- **Subset-A** contains 6 CSV processed data files. The CSV files are generated after processing the corresponding raw data. Each CSV file contains 90 seconds of data recorded by the corresponding receiver.
- **Subset-B** and **Subset-C** respectively contain 20 and 35 CSV processed data files, as result of processing the corresponding raw data. Each processed CSV file contains 2 hours of data collected by the corresponding receiver.

For the MatLab source code (**Code**), we provide 4 main scripts:

- **ProcessMyRawData_ABC.m** is used to process the raw data stored in **Raw-Data** folder, which results in the data stored in **Processed-Data**. In order to execute this script it is necessary the secondary scripts: **dialogselectfolder.m, readrawfiles.m, and bleformat.m;**
- **Disp_distribution.m** displays the distribution of the subsets’ processed data stored in **Processed-Data**;
- **Val_pathloss.m** and **Val_collab.m** provide the technical validations in LOS. Additionally, a **Readme.txt** file is provided to explain in details how to use all scripts.

**Fig. 12:**
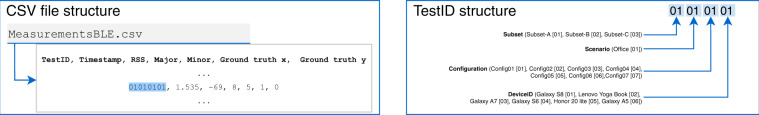
CSV file and TestID structure examples.

The file naming conventions are as follows. For the files with processed data (CSV files), they were all named as **MeasurementsBLE.csv**. Each row of the CSV files is arranged as follows (see Fig. 12):

- TestID: A numerical code, of six digits, used to identify the subset, scenario, configuration, and device used in the data collection.
- Timestamp: The time, in second, at which each devices’ BLE packet (only the ones used in the experiments) is read by the *GetSensorData* app in the receiving mobile device.
- RSS: The received signal strength of the mobile devices used in the experiments, indicated in dBm, measured at the specified Ground-Truth.
- Major: The identifier value used to group the mobile devices’ BLE packets and identify them from others groups.
- Minor: The identifier value used to identify the mobile devices’ BLE packets within the group to which they belong.
- Ground Truth *x*: Indicates the real *x* coordinate, in meters, of the mobile devices within the Office scenario in accordance with each configuration.
- Ground Truth *y*: Indicates the real *y* coordinate, in meters, of the mobile devices within the Office scenario in accordance with each configuration.

For the *logfiles* with raw data (TXT files), the filename naming convention is as follows: it is separated by underscores and is composed of an initial name (“logfile”), the date (yyyy_mm_dd), and time (HH_MM_SS) of the end of the recording. In the file, the data of BLE packets is saved in sequential order of arrival (one row per packet received) and the fields in each row are separated by semicolons. Specifically, the data format for the BLE packets saved is arranged as follows.

- Type of technology: Fixed Identifier (“BLE4”) at the beginning of each row, which denotes the kind of sensor used for measurements.
- Timestamp: The time, in second, at which each devices’ BLE packet (all available devices) is read by the *GetSensorData* app in the receiving mobile device.
- Type of beacon: Indicates the beacon format read (“iBeacon”/“Eddystone”).
- MAC: The Media Access Control (MAC) of every device read by the *GetSensorData* app in the receiving mobile device.
- RSS: The Received-Signal-Strength (RSS) of all surrounding devices, indicated in dBm, measured.
- Transmission Power: Indicates the power of transmission (dBm) of BLE packets.
- Major: The identifier value used to group the devices’ BLE packets and identify them from others groups.
- Minor: The identifier value used to identify the devices’ BLE packets within the group to which they belong.
- UUID: The Universal Unique Identifier (UUID) is a unique number used to identify the devices’ BLE packets.

Further information about the sensor data formats of the *GetSensorData* can be consulted in^15^. As already mentioned, except for including broadcasting BLE advertisements, the other modules of the application remained unaltered with respect to the original version.

## Technical Validation

### Distance estimation based on Path-loss model and signal filtering

This subsection provides a validation of the data collected for Subset-A, i.e., the calibration data, captured under LOS conditions, required to train or create a model that provides the distance estimation between two devices using Received-Signal-Strength (RSS) values as input. In order to use the calibration results of this subsection in the Trilateration approach of the following subsection, we focus this technical validation to the data collected with the receiver Device 02, whose corresponding data can be found in the path **Processed-Data/Subset-A/Office/Config01/ReceiverDev02/MeasurementsBLE.csv**.

We adopted the Logarithmic distance path-loss model^5,21,22^ (see Eq. 1) to determine the distance between the transmitter and receiver from the RSS values.

1 $$RSS(d)=RSS({d}_{0})-10\times \eta \times log\left(\frac{d}{{d}_{0}}\right)$$

where *RSS*(*d*) is the RSS at a distance *d* between transmitter and receiver devices; *RSS*(*d*_0_) is the RSS at a reference distance *d*_0_, which is usually considered at 1 m; *η* is a path-loss attenuation factor. The RSS and distances are expressed in decibels and meters respectively.

**Fig. 13:**
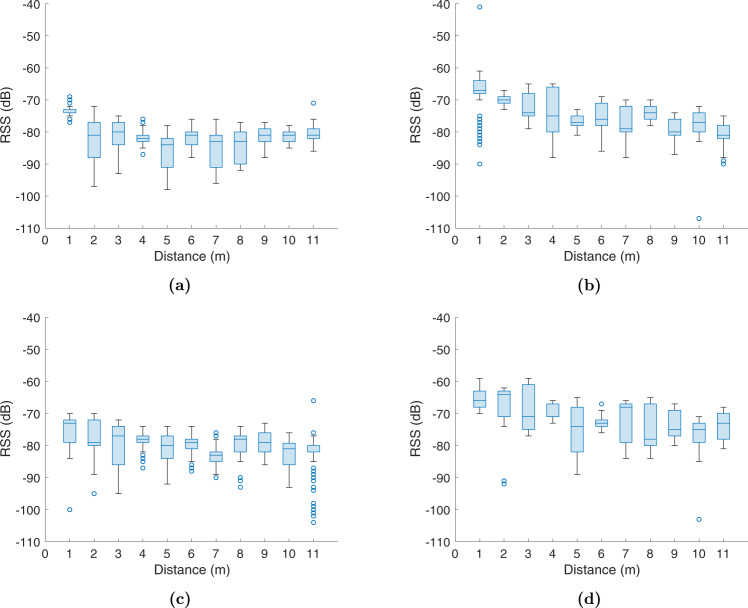
Distribution of the RSS values of the Config01 A collected with the Device 02 (receiver) on each reference point. **(a)** Boxplot of the RSS values considering the Device 01 as Transmitter, **(b)** Boxplot of the RSS values considering the Device 03 as Transmitter, **(c)** Boxplot of the RSS values considering the Device 04 as Transmitter, and **(d)** Boxplot of the RSS values considering the Device 05 as Transmitter.

Additionally, we used the Non Linear Least Squares method with the recorded data to obtain optimal parameters (*RSS*(*d*_0_) and *η*) for the Logarithmic distance path-loss model. The fitting curve provides the relation between the RSS values and geometric distances between devices. However, the nature of RSS measurements, even in LOS conditions, is noisy (see Fig. 13). Before training the model, pre-processing is needed to smooth data and reduce the outliers effects. After filtering the raw data, we average the samples for each reference point.

**Fig. 14:**
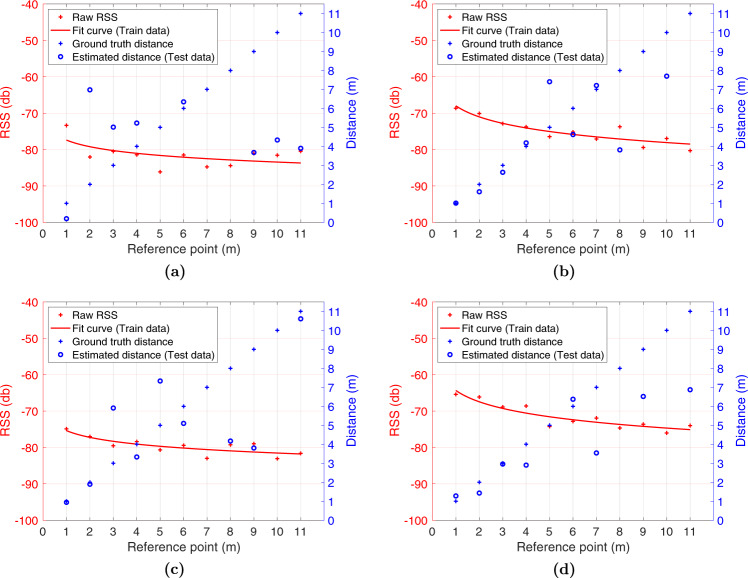
Curve fitting based on Path-loss model and distance estimation using the RSS values, smoothed with a moving average filter, of the Config. 01 A collected with the Device 02 (receiver). **(a)** Results of the Device 01 as Transmitter, **(b)** Results of the Device 03 as Transmitter, **(c)** Results of the Device 04 as Transmitter, and **(d)** Results of the Device 05 as Transmitter.

Figure 14 shows the results of applying a Moving Average filter to the raw RSS values (red crosses) corresponding to the Devices 01, 03, 04, and 05 which act as transmitter in Configuration 01A. The figure also shows the fitting curve (solid red line) for each transmitter device, whose parameters (*RSS*(*d*_0_) and *η*) and the distance error, between the estimated and GT distance, are provided in Table 5. The table also contains the result of applying three additional filters, Moving Average, Moving Median, and Robust Locally Weighted Scatterplot Smoothing (RLOWESS). To evaluate the goodness of curve fit with our data, we provide the Sum Square Error (SSE), Rsquare, and RMSE (Root-Mean-Squared Error) for each transmitter device and filter used. Also, we computed the RMSE, Mean-Squared Error (MSE) and Standard Error of the estimated distances.

**Table 5:** Goodness of curve fit and Distance errors.

ID	Filter	RSSI(d_0_)	*η*	Goodness of curve fit (RSS)			Distance (m)		
				SSE	Rsquare	RMSE	MSE	RMSE	Std. Error
01	Mov. Average	−77.39	0.60	71.04	0.35	2.8	106.76	10.33	10.15
	RLOWESS	−77.48	0.59	67.32	0.35	2.73	103.65	10.18	10.13
	Mov. Median	−76.75	0.64	48.14	0.47	2.31	35.62	5.96	6.11
03	Mov. Average	−67.97	1.01	23.24	0.82	1.6	6.43	2.53	2.64
	RLOWESS	−67.49	1.05	21.68	0.84	1.55	5.7	2.38	2.49
	Mov. Median	−67.43	1.06	22.6	0.84	1.58	7.96	2.82	2.83
04	Mov. Average	−75.36	0.61	19.9	0.66	1.48	20.41	4.51	4.6
	RLOWESS	−75.21	0.61	24.91	0.61	1.66	45.26	6.72	6.83
	Mov. Median	−75.24	0.61	22.49	0.63	1.58	29.4	5.42	5.62
05	Mov. Average	−64.33	1.03	19.84	0.84	1.48	11.3	3.36	3.5
	RLOWESS	−64.45	1.01	22.1	0.83	1.56	17.77	4.21	4.4
	Mov. Median	−64.11	1.03	26.22	0.8	1.7	35.16	5.93	6.08
06	Mov. Average	−65.85	1.37	33.1	0.85	1.91	4.5	2.12	2.2
	RLOWESS	−65.86	1.37	34.99	0.84	1.97	5.16	2.27	2.36
	Mov. Median	−66	1.34	34.96	0.84	1.97	5.46	2.33	2.44

According to the analysis of the results in Table 5, we conclude that the Moving Average filter considering 30 samples slightly out-performs the other filters allowing us to better smooth the RSS and obtain a lower distance error in most of the devices. Furthermore, the path-loss factor is different for each transmitter device, within the range of values 0.59 to 1.37, and the *RSSI*(*d*_0_) is on average −77.2 dBm, −67.63 dBm, −75.27 dBm, −64.29 dBm, and −65.9 dBm for devices 01, and 03 to 06 respectively. Although the RMSE in estimating the distance is large, due to hardware configuration and environment geometries, we observe in Fig. 14(b–d) that under the first 4 meters the difference between the GT and estimated distance is around 1 meter.

### Trilateration approach based on collaborative users

This subsection provides a validation of the data collected for subsets B and C, i.e., the data that enables collaborative positioning (including ranging). Due to the high volume of subsets B and C, we restrict this technical validation to the data collected in the first, second and third collaborative configurations (see Figs. 4–6) and registered with the Device 02. The six files used in this collaborative validation (one per subset and collaborative configuration) are in the database path folder:

- /Processed-Data/Subset-B/Office/Config01/ReceiverDev02/MeasurementsBLE.csv
- /Processed-Data/Subset-B/Office/Config02/ReceiverDev02/MeasurementsBLE.csv
- /Processed-Data/Subset-B/Office/Config03/ReceiverDev02/MeasurementsBLE.csv
- /Processed-Data/Subset-C/Office/Config01/ReceiverDev02/MeasurementsBLE.csv
- /Processed-Data/Subset-C/Office/Config02/ReceiverDev02/MeasurementsBLE.csv
- /Processed-Data/Subset-C/Office/Config03/ReceiverDev02/MeasurementsBLE.csv

We assume a collaborative scenario where five users (User 1 to 5) hold each a device, Device 01 to Device 05 for the Subset-B and Devices 01 to Device 04 and Device 06 for the Subset-C respectively. Users 1 and 3–5 know their exact position (for instance by using a Ultra-Wideband (UWB) positioning solution) and share it (for instance using a centralized platform) to User 2, which is assumed not to be able to self-determine its position using this advanced technology. By using the exact position, the dataset allows collaborative positioning algorithms to be evaluated without the accumulated error estimated positions would introduce. User 2 uses the available information (the position of users 1 and 3–5) and the estimated relative distances to them (using the RSS values and Path Loss model) in order to establish its own location by means of a collaborative method. Even though this collaborative scenario resembles a classical positioning scenario using the Users 1 and 3–5 as regular beacons, the diversity of hardware (at smartphone and Bluetooth chipset levels) and Software (Operating System version and vendor’s customization layer) employed makes this positioning more challenging.

**Fig. 15:**
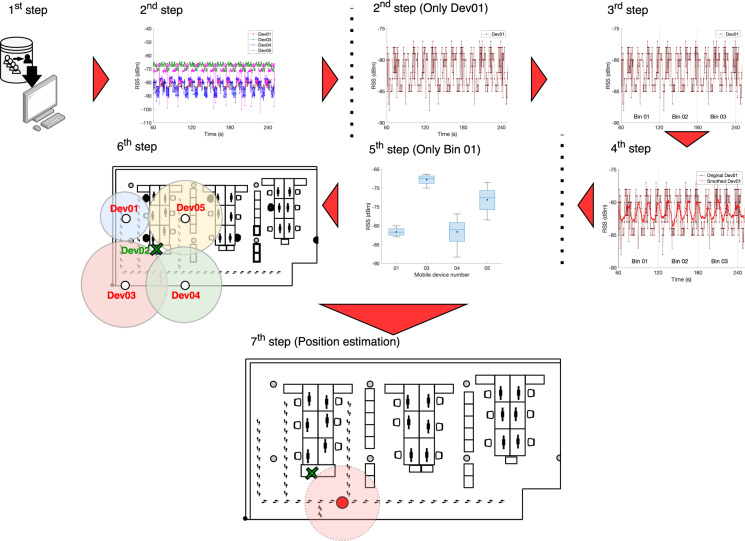
Collaborative positioning approach workflow.

The workflow for the collaborative method is presented in Fig. 15 and is summarized as follows:

- **1**^**st**^ **step:** Select user (i.e. User 2) and load the data his/her device registered from the remaining users (Users 1 and 3–5).
- **2**^**nd**^ **step:** Group the collected RSS values by device (Users 1 and 3–5) and get their position from the centralized platform);
- **3**^**rd**^ **step:** Group the RSS values in bins of 60 s;
- **4**^**rd**^ **step:** Remove the outliers and smooth the noisy data with a moving average of 30 samples;
- **5**^**th**^ **step:** average the RSS of each device contained in each interval of time selected in the first step in order to get only one value for each interval;
- **6**^**th**^ **step:** estimate the distances using the Logarithmic distance Path-Loss (see Eq. 1) and the averaged RSS values from 4th step. We considered the path-loss attenuation factor (*η*) of 0.6, 1.01, 0.61, and 1.03 and the *RSS*(*d*_0_) equal to −77.39 dBm, −67.97 dBm, −75.36 dBm and −64.33 dBm for Users 1 and 3–5 respectively;
- **7**^**th**^ **step:** The position is estimated for User 2 using the Levenberg-Marquardt Least Squares (L-MLS) Trilateration method to fit the Euclidean Distance model. The input data to fit the model are the distances estimated in the fifth step, their weights and the location of Users 1 and 3–5. The weight values for every user are computed as the inverse of their distance square.

The results of the collaborative method are summarized in Table 6, which reports different metrics based on the positioning error of User 2.

**Table 6:** Statistical values of the Euclidean distance error of each Collaborative scenario example.

Subset	Conf.	*Mean* (m)	*Median* (m)	*P*_25_ (m)	*P*_75_ (m)
B	01	1.72	1.75	1.68	1.78
C		0.60	0.61	0.55	0.66
B	02	4.08	3.77	3.54	4.78
C		3.33	3.54	2.23	4.42
B	03	1.87	1.78	1.58	2.07
C		1.02	0.94	0.44	1.47

Summarizing the results, the straightforward collaborative approach provides good positioning estimates in a moderate obstruction of LOS and short distance between collaborative users and target user (i.e., User 2), as was demonstrated with the results of collaborative configuration 01 and 03. However, in case of large and equidistant distances (collaborative configuration 02), the mean of the Euclidean distance error were the biggest, i.e. 4.08 m, for cases with deliberate and frequent walking in the office, which prolonged the obstruction of LOS, and 3.33 m for normal, more moderate walks of workers around the office.

It needs to be mentioned that the applied positioning strategy served to validate the collected dataset; improvements, such as using a dynamic set of weight values and a more aggressive outliers detection strategy in the collaborative approach could improve the robustness and accuracy of the method. However, this is outside the scope of this article.

## Usage Notes

The presented mobile device-based BLE Database can be used for the following applications:

- Calibration of Contact tracing applications. Contact tracing systems based on mobile devices’ BLE signals, which is one of the most used technologies, are prone to inaccuracy mainly due to harsh environment conditions and hardware heterogeneity. In consequence, experimentation and studies towards the calibration and analysis of BLE signal propagation in a real indoor environment and with heterogeneous mobile devices are needed. **Subset-A** provides exactly the data needed to perform such studies.
- Evaluation of Contact tracing. The data collected in **Subset-B** and **Subset-C** can be used to evaluate Contact tracing systems in LOS and NLOS conditions in realistic contexts.
- Calibration of Collaborative IPS. Collaborative Indoor Position System (IPS) based on BLE technologies for collaboration between devices uses the BLE signals to estimate the relative distance between collaborative users. Nevertheless, the heterogeneity of devices used requires a further characterization and analysis of each transmitter and receiver device to enhance the accuracy of the overall collaborative system. This analysis can be performed with the information in LOS provided in **Subset-A**. For instance, the estimation of path-loss model parameters (*RSS*(*d*_0_) and *η*) performed in the Technical validation section. In addition, **Subset-A** can be used to generate or test proposed algorithms focused in parameter unification of heterogeneous mobile devices.
- Evaluation of algorithms and methods for collaborative positioning. **Subset-B** and **Subset-C** contains a rich and documented combinations of non-ideal scenarios of BLE-RSS signal and Ground-Truth (GT) positions, which provide diverse arenas to evaluate the performance of collaborative approaches. For example, the Collaborative algorithms based on trilateration, artificial neural networks, and other machine learning algorithms for indoor positioning.

In addition, the proposed folded-based structure eases the indoor positioning community to extend this dataset by adding new scenarios and configurations. For example, configurations with one or more non-stationary mobile devices moving in the scenario, along with data synchronization between them. A common format in all datasets will ease their adoption by the community and other researchers interested in collaborative positioning. As far as we know, this is the first database in dealing with smartphone-based collaborative indoor positioning, having the potential of becoming a *de-facto* standard for future releases by other research teams.

## Data Availability

The data collection was conducted using the application *GetSensorData*, which is available at the GitLab repository^17^. The processing of the raw data, displaying of the distribution of the subsets and the technical validations of the data collected was done in Matlab. The version required to run the code for processing the raw data is R2017a or above, and for the rest of scripts (display and technical validations) it is R2020a or above. Also, the Statistics and Machine Learning toolbox is required for the functions used in the technical validations. The source code is available at the Zenodo repository^20^.
